# Why Did (Almost) No One See the Inflation Coming?

**DOI:** 10.1007/s10272-022-1034-9

**Published:** 2022-04-08

**Authors:** Jason Furman

**Affiliations:** Kennedy School of Government, Harvard University, 79 John F. Kennedy Street, Cambridge, MA 02138 USA

**Keywords:** E31, E37, E65

## Abstract

To understand the possible trajectory of inflation in 2022 and beyond, it is helpful to understand why the United States and Europe had so much inflation in 2021.

The United States and Europe are both experiencing the fastest inflation in a generation. The inflation in both economies was not foreseen by the standard economic models used by official and private sector forecasters. This failure should lead to some reassessment of the models and should increase uncertainty and concern about the trajectory of inflation going forward. In particular, policymakers should not rely on statistical relationships that held in the decades before the pandemic when making predictions in today's very different environment. This situation calls for both rethinking the underlying economics – for example, inflation will play a more salient role in setting wages and prices at its faster pace so wage-price passthrough could be higher as well – and widening confidence intervals to reflect the greater uncertainty.

Specifically, the linear Phillips curve with anchored expectations failed to predict the inflation of 2021 because, by construction, that Phillips curve can essentially never predict high inflation. Even with a massive fiscal stimulus that cut the unemployment rate to the likely impossibly low level of 1%, the inflation rate would still be predicted to remain below 3%. An alternative model, in which fiscal stimulus predicts nominal (not real) demand, real output can rise but not above its short-run potential, and inflation is the difference between the two, does a much better job of making sense of the extraordinary inflation in 2021 by dispensing with the labor market intermediation.

Despite the shared underestimation of inflation, the specific situations differ on the two sides of the Atlantic with inflation running considerably higher in the United States than in Europe and the GDP recovery conversely further behind in Europe. Policy in Europe should avoid the trap of being too driven by developments and news in the United States.

* Wilson Powell III provided outstanding research assistance for this piece.

**Jason Furman**, Harvard University; and Peterson Institute for International Economics, Washington DC, USA.

## What were the major forecasters thinking when they predicted low inflation?

To understand the possible trajectory of inflation in 2022 and beyond, it is helpful to understand why the United States and Europe had so much inflation in 2021. This analysis is based on the U.S. experience; many of the same lessons and takeaways also apply in the European context – albeit all of them to a lesser degree.

None of the major private sector or official sector forecasters saw the 2021 inflation coming nor was it reflected in market prices, as shown in Table 1. All of the forecasters appeared to be using relatively standard multipliers to analyze the impact of the fiscal expansion on *real* GDP and then a relatively flat, linear Phillips curve with anchored expectations to simulate the impact of real GDP and unemployment on inflation. Given that estimates of the slope of the Phillips curve vary from about 0.1 to about 0.3, it is *impossible* to generate much inflation from this setup. Even if the unemployment rate had been driven down to the historically, and likely impossibly, low 1%, and the natural rate was 4%, the result would only have been 2.3% to 2.9% inflation. A linear Phillips curve with anchored expectations simply cannot explain the inflation of 2021 nor can it explain the variations in inflation rates over time and across countries, which are considerably larger than any differences in slack could justify.

Working through a typical multiplier model, we can understand why the linear view of the world produced the predictions it did. Figure 1 shows the GDP forecast made in December 2020 by IHS Markit, a leading forecast firm. It then adds to it based on the $2.8 trillion of fiscal assistance passed in December 2020 and March 2021 two sets of multipliers: normal multipliers that were used in the past by the Council of Economic Advisers (CEA; 2009, 2014) and low multipliers, which are suppressed by the non-pharmaceutical interventions associated with COVID-19, from the Congressional Budget Office (CBO; 2020a).[1] Figure 2 mechanically translates the GDP numbers into unemployment numbers based on the relationship between GDP growth and employment gains described by the Council of Eco-

1 The cumulative four-quarter normal multipliers are: 1.44 for public investment outlays, 0.66 for individual tax cuts, 0.98 for state fiscal relief, 1.44 for aid to directly impacted individuals and 0.08 for business tax incentives. The cumulative four-quarter low multipliers are 0.66 for enhanced unemployment, 0.44 for recovery rebates, 0.59 for direct assistance to state and local government, 0.07 for business tax provisions and 0.27 for the Paycheck Protection Program.

Table 1
### 2021 (Q4/Q4) inflation forecasts

| | Measure | Q1 | Q2 | Q3 | Actual |
|---|---|---|---|---|---|
| Survey of Professional Forecasters | Core PCE | 1.8 % | 2.3 % | 3.7% | 4.6 % |
| Federal Open Market Committee | Core PCE | 2.2 % | 3.0 % | 3.7% | 4.6 % |
| Congressional Budget Office | Core PCE | 1.5 % | | 2.4% | 4.6 % |
| Federal Reserve Bank of New York DSGE | Core PCE | 1.4 % | 2.2 % | 3.8% | 4.6 % |
| OECD | Core CPI | | 3.0 % | | 5.0 % |
| International Monetary Fund | CPI* | | 2.3 % | | 7.0 % |
| Market-based | CPI | 2.7 % | 2.9 % | | 6.7 % |

Notes: *International Monetary Fund forecast is Dec./Dec. DSGE: dynamic stochastic general equilibrium, OECD: Organisation for Economic Co-operation and Development, PCE: personal consumption expenditure, CPI: consumer price index. Core measures exclude food and energy.

Source: Organizations listed; author's calculations.

Figure 1
### Estimated effect of December 2020 and March 2021 fiscal stimulus on real GDP
in billion chained 2012 U.S. dollars

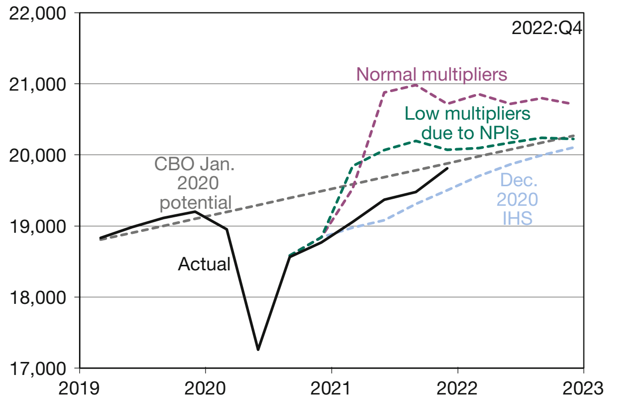

Notes: NPIs: non-pharmaceutical interventions. Normal multipliers based on CEA (2009, 2014); low multipliers based on CBO (2020a).

Source: CBO; IHS Markit; CEA; Bureau of Economic Analysis, Macrobond; author's calculations.

Figure 2
### Estimated effect of December 2020 and March 2021 fiscal stimulus on the unemployment rate
in percent

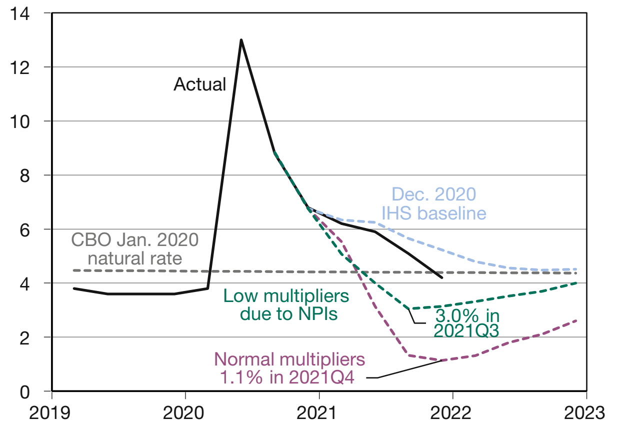

Notes: NPIs: non-pharmaceutical interventions. Normal multipliers based on CEA (2009, 2014); low multipliers based on CBO (2020a).

Source: CBO; IHS Markit; CEA; Bureau of Labor Statistics, author's calculations.

nomic Advisers (2009). In the case of the normal multipliers, this results in the economically absurd forecast of a 1.1% unemployment rate in the first quarter of 2021, a sign that something is wrong with this methodology – a topic I will return to.[2]

Even the implausibly low unemployment rate would not have been expected to translate into much inflation using a conventional approach. Ball et al. (2021), for example, estimate that the Phillips curve has a slope of -0.17 – i.e. each one percentage point reduction in the unemployment rate boosts the inflation rate by 0.17 percentage points. Figure 3 shows predicted inflation based on this Phillips curve. (Note, a Phillips curve approach cannot explain the U.S.-euro area inflation differential and in fact would predict *higher* inflation in Europe because employment was higher there.)

In summary, forecasters using major models should have been nervous that absent any judgmental adjustments, their models were forecasting GDP well above potential and implausibly low unemployment rates. Regardless of such judgmental adjustments, however, the models still would not have predicted much inflation – because no amount of unemployment rate reduction can generate much inflation from these models.

2 In reality, no forecasters predicted an unemployment rate like this and in fact virtually all of them expected the unemployment rate to still be above the pre-COVID-19 rate by the end of 2021. Most forecasters, however, did expect GDP to be above its pre-pandemic trend by the end of 2021. The difference was bridged by implicit or explicit forecasts of a temporarily very large increase in productivity growth – the residual between GDP growth and employment growth. IHS Markit's forecast for 2021Q4 GDP, for example, increased by 5.4% between December 2019 and June 2021.

Figure 3
**Estimated effect of December 2020 and March 2021 fiscal stimulus on core CPI inflation**
four-quarter percent change

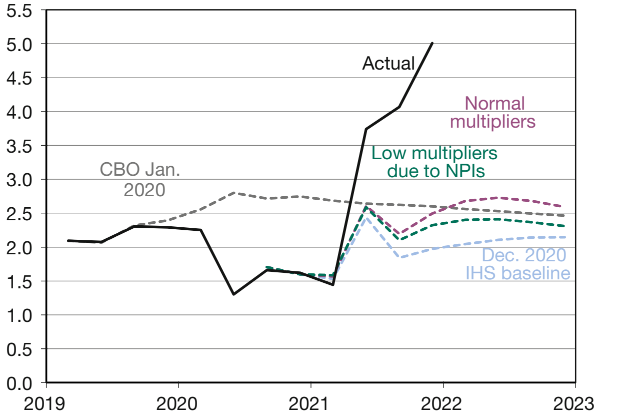

Notes: NPIs: non-pharmaceutical interventions. Normal multipliers based on CEA (2009, 2014); low multipliers based on CBO (2020a).

Source: CBO; IHS Markit; CEA; Ball et al. (2021); Bureau of Labor Statistics; author's calculations.

## Where did all the inflation come from if not from a linear Phillips curve?

The general Phillips curve is:

*Inflation = expected inflation – θ*(unemployment – natural rate of unemployment) + error term*

The discussion of the increase in inflation is organized around the different terms of this equation.

### A positive error term: Supply shocks and COVID-19 taste changes

One possibility is that the inflation we have seen reflects the error term – unforeseeable events that happened essentially outside the economic model and did not transmit to inflation through aggregate demand or the labor market. It is likely that is part of the story but probably only part of the story. Some of the main candidates for the error term include:

*The emergence of the Delta and Omicron variants of COVID-19*. Slowing the reopening of the economy is commonly cited as a reason that inflation was higher than expected in the second half of 2021. But the rapid reopening of the economy as people were vaccinated in the first half of the year was also cited as a reason for rapid inflation then. While it is possible both arguments were true, it seems unlikely. The resurgence of COVID-19 likely raised durable goods prices but lowered service and gasoline prices. The net effect is ambiguous but evidence from earlier in the pandemic suggests this was more plausibly negative than positive because, first, the initial wave of COVID-19 in 2020 reduced inflation. Second, the timing of the inflation generally followed the reopening of the economy, rising first in the United States when its economy was reopening and then later in the euro area when its economy reopened somewhat later. This suggests that the net effect of the coronavirus pandemic is to suppress inflation and that inflation would have been even higher without the Delta and Omicron variants. Even if not correct, it is unlikely that the Delta and Omicron variants had a large positive effect.

*The shift from services to goods*. Another candidate for the error term is what could be described as a taste shock: For example, people felt unsafe in the gym so, instead of paying gym memberships, they bought exercise bicycles. To the degree that the supply of goods is more inelastic than the supply of services, this would increase inflation. There are two issues with this theory, however. First, the increase in goods spending seems more a consequence of the overall level of demand than a taste shift – goods spending was considerably higher in the spring of 2021, as COVID-19 case numbers were low and falling, than it was in the winter of 2020-21, when case numbers were high and rising. Moreover, both goods and services spending was higher in the United States than in Europe (although service spending in the two economies had largely converged by the end of 2021). This suggests that it was the economic impact payments and other fiscal support that drove goods spending not a taste shift. Second, while it is plausible that the supply curve for goods is more inelastic than the supply curve for services, there still would have been some additional services inflation if there had been less of a shift from goods to services. As a result, the goods-services shift is at most part of the story of the error term.

*Supply chain disruptions*. There is no doubt that supply chain disruptions explain some of the increase in inflation, most notably in microchips and the dynamics of rental fleet purchases and sales of used vehicles. These drove the spectacular increase in motor vehicle and parts prices that contributed 1.1 percentage points to core personal consumption expenditure (PCE) inflation in 2021. But a lot of the so-called supply chain issues are really large increases in demand coming up against supply that was relatively inelastic. The result was a combination of higher prices and higher quantities. U.S. ports, for example, were not disrupted and, in fact, were processing 18% more than in 2019. This, however, was not enough to keep up with demand – as a result, both prices and quantities increased.

*The "great resignation" as labor force participation remains low*. This is a U.S.-specific factor of low labor force participation. It may have played a role in increasing inflation by reducing supply. But it also decreased demand so the net effect on inflation is unclear and unlikely to be very large. Also, the effects of the great resignation on inflation depend on interactions with other fiscal support. It is possible that this could have had more of a role in the early part of 2021 since many people who were not working could get unemployment insurance sufficient to maintain their consumption until September 2021. Now, however, people returning to work are likely to increase both supply and demand.

### A steeper Phillips curve or tighter labor markets

Part of the disconnect between predicted and actual inflation may be related to the slack term in the Phillips curve. Some plausible modifications could add at most about one percentage point to inflation, bridging part of the gap, but not all of it.

*A steeper Phillips curve*. It is possible that low unemployment translates into a larger increase in inflation than the 0.17 percentage point assumed above based on Ball et al. (2021). There are a number of difficulties in estimating the slope of the Phillips curve and more effective monetary policy and anchored inflation expectations can create the statistical illusion that the Phillips curve is flatter than it seems. Hazell et al. (2022) get around these issues by using state-level data on the relationship between unemployment and inflation finding a coefficient that is closer to 0.3. Even this, however, cannot generate much inflation, certainly nothing like the roughly 5% inflation the United States has been experiencing.

*A temporarily higher natural rate or a speed limit*. Even if the natural rate of unemployment was 3.5% in the run-up to the pandemic, it was likely higher during 2021, especially the first half of the year as it takes time for people to connect to jobs, hysteresis temporarily raises the natural rate, the pandemic itself temporarily disrupted people from taking jobs, and unemployment insurance reduced the willingness of people to take jobs. Alternatively, it is possible that there is a "speed limit" of how fast employment can improve without triggering inflation (which could be modelled as a temporarily higher natural rate that only falls slowly; Turner, 1995). These changes, however, could not add much to inflation because they are limited by the relative flatness of the Phillips curve itself – even a 5% natural rate combined with a 0.3 slope of the Phillips curve would generate less than an additional 0.5 percentage point of inflation.

*Alternative measures of slack showed a tighter labor market*. The unemployment rate is the standard measure of

Figure 4
**Inflation expectations**
in %, annual rate

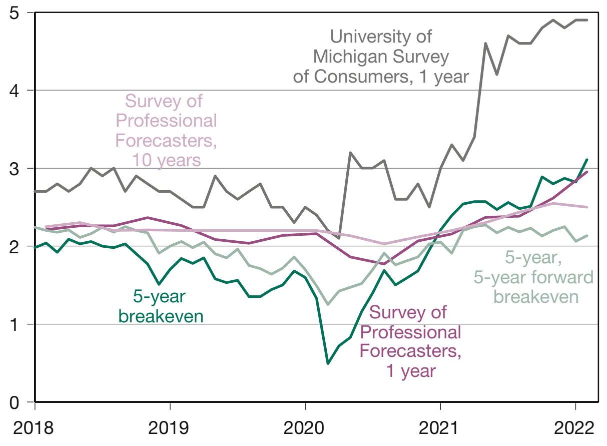

Source: Federal Reserve Bank of Philadelphia, Survey of Professional Forecasters; Macrobond; University of Michigan; author's calculations.

slack. But in 2021, job openings and the quits rate both soared, suggesting a much tighter labor market than indicated by the unemployment rate, especially earlier in the year. Some evidence suggests that the ratio of unemployed to job openings and quits are both better predictors of inflation than the unemployment rate; using them would add more to the inflation prediction (Furman and Powell, 2021; Domash and Summers, 2022).[3]

### Higher expected inflation

The final possible explanation for the recent inflation, sticking with the linear Phillips curve model described at the start of this section, is that expected inflation increased. This is also not a satisfying explanation because any increases in expected inflation mostly *followed* the price and wage increases instead of preceding them and the increases in expectations were relatively small. At the end of 2020, financial market expectations for inflation over the next five years were very low, they rose sharply starting in December 2020 but settled only modestly above their normal level (see Figure 4). Consumers did increase their near-term inflation expectations sharply starting in January 2021. And, as shown in Table 1, forecasters were well behind inflation all year. Moreover, most forecasting models incorporate long-run, not short-run, inflation expectations and those

3 In the prediction models reported by Furman and Powell (2021), they find the adjusted $R^2$ in predicting core CPI is 0.47 for quits as an explanatory variable, 0.45 for the ratio of unemployed to job openings, 0.35 for the unemployment rate and 0.22 for the prime-age (25-54) employment rate.

remained well anchored all year (Reifschneider and Wilcox, 2022).

## Is the Phillips curve nonlinear?

Looking at reasonable ranges for the parameters of the linear Phillips curve above, it is possible to make changes that generate some additional inflation but they do not plausibly account for all of the inflation in 2021. Moreover, many of these changes are ad hoc and may not actually be right. And none of them provide a particularly satisfying explanation.

To understand the limits of the linear Phillips curve for this situation, consider a much more extreme policy. Imagine that households were each given $100,000 in 2019. An economist using a linear Phillips curve would not predict very much inflation because the policy could not lower the unemployment rate below 0% and so the tight labor market (the only way the Phillips curve incorporates demand) would not add much more than one percentage point to the inflation rate. But clearly a forecast that this policy would lead to only 3% inflation is absurd.

The better way to think about this thought experiment – and the less extreme actual policy carried out in 2020 and 2021 – is through a highly nonlinear model. There is some evidence that the Phillips curve itself is nonlinear (e.g. Nalewaik, 2016; Fair, 2021; Forbes et al., 2021). But a lot of other research has found that any nonlinearities in the Phillips curve are not robust or are unstable and that it is better to work with a linear one (e.g. Marcellino, 2008). Moreover, even a standard nonlinear Phillips curve would struggle with the fact that we have never before seen core PCE inflation jump in this way despite the unemployment rate being well within its normal range. Additionally, it is not a particularly satisfying way to generate *ex ante* predictions – the nonlinear Phillips curve would not be a good way to predict the differences in inflation that would result from increasing the hypothetical helicopter drop from $100,000 to $1 million per household.

A better model is to dispense with the additional step of modelling the impact of demand on the labor market and the labor market on inflation and instead just go straight from nominal demand to inflation.

## Fiscal stimulus as supporting nominal demand, not real demand

Most of the microeconomic research that has been used to develop fiscal multipliers actually looks at parameters like the marginal propensity to consume in nominal terms – asking questions like, "If you give people $100, how much do they increase their nominal spending?" (e.g. Parker et al., 2013; Sahm et al., 2012). This suggests a simple three-step framework for thinking about inflation in 2021:

Figure 5

**Multiplier estimates vs pre-pandemic potential**

in % of CBO January 2020 potential real GDP

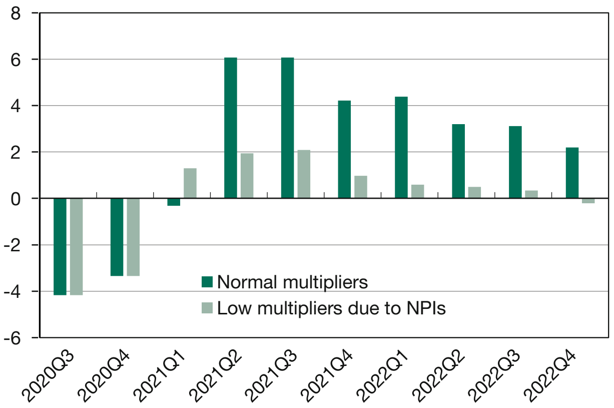

Notes: NPIs: non-pharmaceutical interventions. Normal multipliers based on CEA (2009, 2014); low multipliers based on CBO (2020a).

Source: CBO; IHS Markit; CEA; author's calculations.

1. Use multipliers to predict *nominal* GDP.

2. Use the productive capacity of the economy adjusted down for the effects of the pandemic to predict *real* GDP. That is, assume that there is a limit on the amount that fiscal support can increase real production.

3. Price increases are the residual.

What this approach means for U.S. inflation in 2021 can be discerned from looking at the prediction the standard multiplier models had for output relative to potential (see Figure 5), which is just another way of showing the results of the multiplier exercise reported in Figure 1. This shows that output was projected to be about 1% to 4% above the pre-pandemic projection of potential in 2021Q4, depending on the multipliers. Even hitting the pre-pandemic projection for potential would have been hard given that the population was smaller due to reduced immigration and excess deaths, the capital stock was smaller due to foregone investment, the COVID-19 pandemic was still disrupting production, and U.S. income support policies like unemployment insurance and stimulus checks caused sustained reductions in labor supply. On the other

Figure 6
**Real gross domestic product**
Index, 2019Q4=100

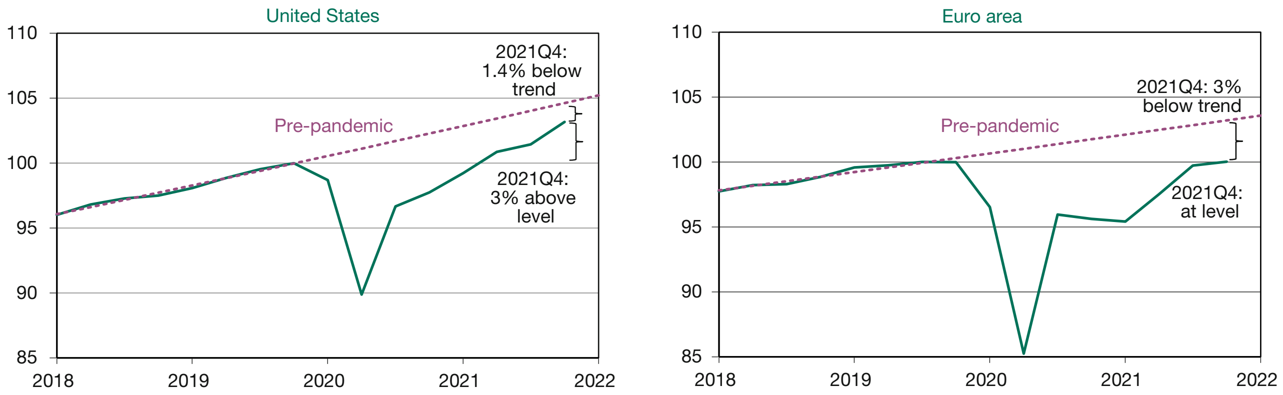

Note: Pre-pandemic trend based on log-linear regression for 2018Q1 to 2019Q4.

Source: Bureau of Economic Analysis; Eurostat; Macrobond; author's calculations.

hand, it is plausible that pre-pandemic expectations for potential were about one percentage point too low given that they assumed the natural rate of unemployment was 4.4% while 3.5% was completely plausible (CBO, 2020b). Accounting for these offsetting effects, assuming the economy was capable of producing at the level of the pre-pandemic projection of potential was a plausible but likely upper bound assumption – leaving about one to four percentage points of additional inflation above the baseline expectation for inflation.[4]

## What does this mean for the outlook for inflation?

Modelling nominal demand and real supply is less useful for forecasting inflation in 2022 and 2023. It is almost certainly the case that reduced fiscal and monetary support will slow nominal demand growth. But it is also almost certainly the case that with the economy close to its potential real GDP, growth will slow too.

4 Two caveats are in order. Baseline expectations for inflation might have been below 2% absent the two rounds of fiscal support in December 2020 and March 2021. IHS Markit, for example, was expecting core PCE to be 1.8% for 2021 Q4/Q4 in its December 2020 baseline. On the other hand, it is possible people had enough excess savings from the transfers in 2020 and the reduced consumption in that year to finance an above-normal level of spending when the pandemic receded and that giving them additional money in this context would have a very low marginal propensity to consume. In this case, the multiplier might be towards the low end but the underlying baseline inflation it would be adding to could be towards the high end.

In the context of the linear Phillips curve, low levels of labor market slack suggest more inflation in 2022 than in 2021 but the error term (e.g. supply shocks), which likely added to inflation could move to zero or even negative in 2022. This would cause inflation to fall relative to 2021.

The trickiest issue to assess is the inflation expectations term. Reifschneider and Wilcox (2022) model this term as largely based on professional forecasts of inflation over the next ten years, which have been stable. But in the current context, short-run inflation expectations may be more relevant and appear to be becoming embedded in wage and price setting (Furman, 2022).

All in, core inflation is likely to be lower in 2022 than it was in 2021. However, with several forces pushing inflation higher, it may still end up in the 3.5% to 4.5% range, depending on the measure used. Moreover, it is plausible that inflation in 2023 will exceed inflation in 2022 if, for example, there is an unusually large one-time decline in goods prices in 2022 due to a glut in the supply of cars.

## Similarities and differences between the United States and Europe

The European situation is somewhat different from the U.S. one because GDP growth has been weaker and inflation has not increased as much, with the apparently smaller European fiscal response likely at least partially responsible for the difference.

Figure 7
**Disposable personal income relative to trend**
Percent difference from trend

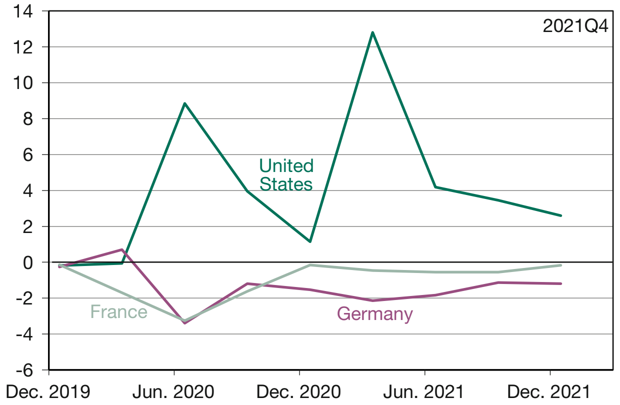

Notes: Pre-pandemic trend based on log-linear regression for 2018Q1 to 2019Q4. Disposable personal income for the United States, adjusted disposable income of households (including sole proprietorships) for France, and disposable income of households (expenditure concept) adjusted for the change in pension entitlements for Germany.

Source: Bureau of Economic Analysis; Statistisches Bundesamt; INSEE; Macrobond; author's calculations.

Figure 8
**HICP inflation**
24-month percent change, annual rate

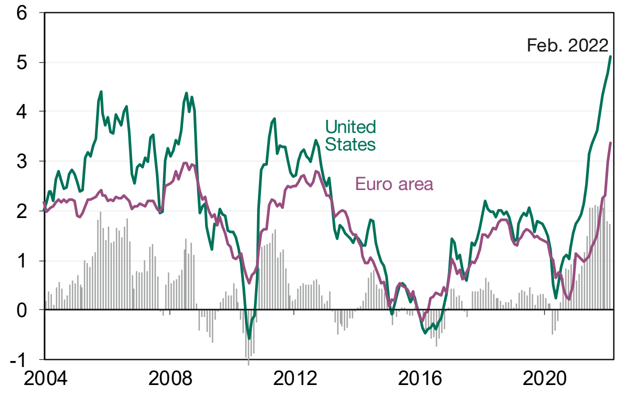

Note: HICP: Harmonised Index of Consumer Prices; estimate for January and February 2022 for the United States.

Source: Eurostat; Bureau of Labor Statistics; Macrobond; author's calculations.

Both the United States and the euro area suffered rapid reductions in GDP when the COVID-19 pandemic hit, followed by partial bouncebacks as restrictions eased. The United States, however, has had a stronger GDP recovery, both in absolute terms and relative to its pre-pandemic trend, as shown in Figure 6. This stronger U.S. GDP recovery has materialized despite U.S. employment lagging employment in Europe as the United States has experienced a large withdrawal and only partial return to the workforce. The gap between these is made up for by the increase in average work hours and a temporary boost in productivity in the United States relative to Europe.

It is difficult to make a meaningful comparison of the size of the U.S. and European fiscal responses because of differences in how fiscal stimulus is described and measured. Ex post deficits and debt are also of limited use, especially when, for example, Germany's deficit numbers appear to reflect macroeconomically unmeaningful charges that increase the deficit and debt in 2021 to make it easier to satisfy the debt brake in future years. The degree to which the U.S. fiscal support was considerably larger than European fiscal support can be seen by comparing the trajectory of disposable personal income in the major economies. Germany and France successfully protected disposable personal income, keeping it close to its previous trend even amidst a massive economic contraction, but that was nothing compared to the huge increase in disposable personal income in the United States, as shown in Figure 7. Other aspects of stimulus may also have been larger in the United States, which, for example, had a considerably larger and less targeted grant program for small and medium-sized businesses, called the Paycheck Protection Program, than anything in the major European economies. The result of the increase in disposable personal income in the United States is that U.S. consumption, particularly of goods, greatly outpaced European consumption.

The flip side of the larger fiscal support and faster pace of U.S. GDP recovery has been higher inflation in the United States, as shown in Figure 8. The United States and Europe have been hit by different supply shocks. The increase in the price of used cars is a bigger deal in the United States, where they are a larger part of the consumption bundle, but Europe has been hit by much larger increases in spot natural gas prices. Europe also had a lower inflation rate in 2020, in part because of the way that temporary value added tax reductions fed into the inflation rate, and experienced larger base effects as its economy moved towards normalizing in 2021. Overall, U.S. core inflation is well above its 2% target trend, whereas the core harmonized index of consumer prices (HICP) in

Figure 9
**Federal funds rate and policy rules**
in %

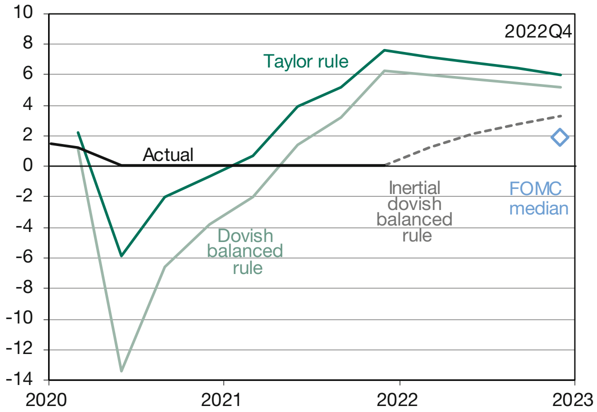

Notes: The figure shows three versions of a Taylor-style rule. The "Taylor rule" uses the Taylor (1993) rule's weight of 0.5 on inflation and 0.5 on the output gap, a natural real federal funds rate of 0.5% and a natural rate of unemployment of 4.0%. The "Dovish balanced rule" raises the weight on the output gap to 1.0, lowers the natural real federal funds rate to 0.0% and the natural rate of unemployment to 3.5%. The "Inertial dovish balanced rule" is the same as the "Dovish balanced rule", only it places 20% weight on this formula and 80% weight on the value of the federal funds rate in the last period. Inflation and unemployment projections based on March 2022 Federal Open Market Committee Summary of Economic Perspectives.

Source: Federal Reserve Bank of New York, Board of Governors of the Federal Reserve System, Bureau of Labor Statistics and Bureau of Economic Analysis via Macrobond; Federal Reserve Bank of Cleveland; author's calculations.

the euro area still falls slightly short of 2% annual growth since the start of the pandemic.

## How should the United States and Europe respond?

Predicting inflation is hard, understanding what to do about it is even harder. The Federal Open Market Committee's expectations for its own interest rate path are much more moderate than even a very dovish version of a Taylor-type rule would imply, as shown in Figure 9. This may well be the appropriate expectation for policy given the many uncertainties in the real economy and financial market reactions, the rapidly diminishing fiscal support for the economy and the desire to avoid risking a recession. But it is very far from the way policy has ever been conducted before.

Europe is closer to its inflation target and further away from its output target. Moreover, Europe faces a potentially much more serious economic impact from the Russian invasion of Ukraine. As a result, it makes sense for the European Central Bank to be more patient in tightening monetary policy, giving the economy more room to recover and more cushion against spillover from the Russian invasion.

While getting inflation under control and keeping expectations anchored is critical in both economies, central bankers also need to be thinking about changing the inflation target itself. Given the decline in equilibrium interest rates, a higher target, like 3%, would give more room for policymakers than the current 2% one. It is possible that the current moment will turn into an opportunity to achieve this new target. But even keeping inflation to 3%, especially in the United States, will be a challenge.
